# Supplementary material for: Risk factors in adolescents as predictors of arterial hypertension in adults: Protocol for a systematic review
Source: PLoS One. 2021 Aug 20;16(8):e0256596. doi: 10.1371/journal.pone.0256596 (PMC8378715; doi:10.1371/journal.pone.0256596)
Supplement: Attachment — Submitted filename: Response to editor.docx [file pone.0256596.s002.docx]

Dear, Anna Mentsi

In response to the considerations made regarding this manuscript being a re-presentation of the manuscript PONE-D-20-35141, I clarify that the manuscript was submitted with the wrong “type of article”. In this first submission he was wrongly classified as:

**Registered Report Protocol** - A Registered Report Protocol is an article that reports a study’s rationale, proposed methodology for data collection and analysis; it may also include pilot data that motivate the research proposal. Registered Report Protocols undergo peer-review prior to conducting experiments, data collection or analysis to ensure that the planned research will meet the PLOS ONE publication criteria. If that is the case, the Registered Report Protocol is published and authors are given an in-principle acceptance in PLOS ONE for the Registered Report article which will report their methods and findings upon completion of the research.

For this reason being disqualified.

On February 10, I received a new email informing that currently the study protocol publication option would be available and open for submissions which led to this new submission.

Attached the rejection e-mail of the manuscript PONE-D-20-35141 and the e-mail of February 10 informing of the new option for publishing protocols

Regards,

Márcia Gisele Santos da Costa
