# Supplementary material for: Risk factors in adolescents as predictors of arterial hypertension in adults: Protocol for a systematic review
Source: PLoS One. 2021 Aug 20;16(8):e0256596. doi: 10.1371/journal.pone.0256596 (PMC8378715; doi:10.1371/journal.pone.0256596)
Supplement: Attachment — Submitted filename: Response to Reviewers.docx [file pone.0256596.s003.docx]

**Response to Reviewers**

1. Please ensure that your manuscript meets PLOS ONE's style requirements, including those for file naming. The PLOS ONE style templates can be found at

<https://journals.plos.org/plosone/s/file?id=wjVg/PLOSOne_formatting_sample_main_body.pdf> and https://journals.plos.org/plosone/s/file?id=ba62/PLOSOne_formatting_sample_title_authors_affiliations.pdf

**Response: done**.

2. Please review your reference list to ensure that it is complete and correct. If you have cited papers that have been retracted, please include the rationale for doing so in the manuscript text, or remove these references and replace them with relevant current references. Any changes to the reference list should be mentioned in the rebuttal letter that accompanies your revised manuscript. If you need to cite a retracted article, indicate the article’s retracted status in the References list and also include a citation and full reference for the retraction notice.

**Response: reference 4 corrected.**

3. We note that you have stated that you will provide repository information for your data at acceptance. Should your manuscript be accepted for publication, we will hold it until you provide the relevant accession numbers or DOIs necessary to access your data. If you wish to make changes to your Data Availability statement, please describe these changes in your cover letter and we will update your Data Availability statement to reflect the information you provide.

**Response: Please make the following change regarding the availability of data: The results of this study will be available in article format to be submitted for publication.**

4. Please include captions for your Supporting Information files at the end of your manuscript, and update any in-text citations to match accordingly. Please see our Supporting Information guidelines for more information: http://journals.plos.org/plosone/s/supporting-information.

**Response: done (page 4, line 92 and after references).**

**Reviewer #1:**

This manuscript contains a plan to review literature to identify childhood predictors of adult hypertension. However, no data was presented. I am unclear as to the rationale for submitting this preliminary work for publication given that there are no results and nothing meaningful will be contributed to the published literature on this subject.

**Response written by the editor**: **Authors first submit a Study Protocol that should include a study’s rationale, its timeline, and proposed methodology for data collection and analysis; this article type does not include results, although it may report pilot data. The authors may disregard reviewer 1’s comments regarding lack of results.**

**Reviewer #2**:

Pg 1 – Please explain numbers 1 and 3 in the affiliation - for the first and last authors.

**Response: We apologize for the typo. The first and last authors are from the same institution. Affiliation has been corrected (number 3 has been removed and inserted number 1 for last author)**

Pg 2- Background – P 2 – “HBP is defined by sustained blood pressure (BP) levels above systolic 140 or diastolic 90 mmHg (millimeters of mercury), or both.” – Please review the definition.
In accordance with most major guidelines it is recommended that hypertension be diagnosed when a person’s systolic blood pressure in the office is ≥ 140 mm Hg and/or their diastolic blood pressure is ≥ 90 mm Hg. This definition does not apply to the United States. In the 2017 ACC/AHA guideline, hypertension is defined as BP ≥130 and / or ≥80 mm Hg.
Another important thing is that these definitions apply to adults ≥ 18 years of age.
For the adolescents, what definition do you intend to use?

**Response: We use the definition of HBP according to the 7th Brazilian Guidelines for Hypertension for adults (included in the text). For the adolescents, in this review we will identify the definition used by the authors of each selected study.**

Pg 5 - If the included population consists of adolescents aged between 12 to 17 years and the outcomes are measured at adulthood (age over 19 years), how are considered subjects aged 18 years – adolescents or adults?

**Response: This has been corrected as the subjects aged 18 years will be considered adults (included in line 104 page 5).**

Pg 5 row 108 – a bracket is missing

**Response: corrected**

Pg 5 row 113 – a space and a comma in addition

**Response: corrected**

Pg 5 – Search methods – “The databases to be searched will include Embase, LILACS, ADOLEC, MEDLINE, Cochrane Library, and bibliographic citations. “ What will be the selection criteria for “bibliographic citations” ?

**Response: Bibliographic citations refers to the references provided in the articles that meet the inclusion criteria. Corrected in the text.**

Pg 5 - There may be too many search terms.
I recommend not using the term “skin pigmentation”; this is not a risk factor for the purpose of this review.

**Response: We agree and removed this term from the search.**

Pg 7 – “The initial deadline for completion is April 2021.” A new deadline must be set.

**Response: Amended to October**
